# Mitochondrial ATPase Subunit 6 and Cytochrome B Gene Variations in Obese Turkish Children

**DOI:** 10.4274/jcrpe.1601

**Published:** 2014-12-05

**Authors:** Durkadın Demir, Doğa Türkkahraman, Anıl Aktaş Samur, Güven Lüleci, Sema Akçurin, Özgül M. Alper

**Affiliations:** 1 Akdeniz University Faculty of Medicine, Department of Medical Biology and Genetics, Antalya, Turkey; 2 Antalya Education and Research Hospital, Clinic of Pediatric Endocrinology, Antalya, Turkey; 3 Akdeniz University Faculty of Medicine, Department of Biostatistics and Medical Informatics, Antalya, Turkey; 4 Akdeniz University Faculty of Medicine, Department of Pediatric Endocrinology, Antalya, Turkey

**Keywords:** Mitochondrial ATPase-6, mitochondrial cytochrome b, obesity, single nucleotide polymorphism

## Abstract

**Objective:** Due to the importance of energy metabolism in mitochondria, mitochondrial genome variations are evaluated in energy-related diseases such as obesity. To date, several nuclear genes were found to be related to obesity. Our aim in this study was to investigate the presence of polymorphisms in mitochondrial ATPase subunit 6 (mt-ATP6) and cytochrome b (mt-CytB) genes that may be associated with childhood obesity.

**Methods:** The mt-ATP6 and mt-CytB genes were amplified and entirely sequenced in a series of 100 obese and in an equal number of healthy Turkish children aged between 6-14 years.

**Results:** A total of 118 synonymous and nonsynonymous variations were detected in the obese and control groups. Only two previously reported synonymous substitutions (mt.8614T>C and mt.8994G>A) in the mt-ATP6 gene were found to be significantly higher in the obese group compared to the control group (p<0.05). In the mt-ATP6 gene, one novel nonsynonymous substitution (mt.8726C>T) and one novel synonymous substitution (mt.9108A>T) were found. In the mt-CytB gene, one nonsynonymous substitution (mt.14880T>C) and two synonymous substitutions (mt.14891C>T and mt.15091C>T) were novel substitutions.

**Conclusion:** Two synonymous substitutions (mt.8614T>C and mt.8994G>A) in the mt-ATP6 gene may be associated with childhood obesity. Our study provides the first data about mitochondrial genome variations in a Turkish obese population and also the first in obese children. More cases should be screened in obese groups in order to understand the effects of mitochondrial polymorphisms in the development of obesity.

## INTRODUCTION

Obesity (OMIM# 601665) is a well-known multifactorial disease characterized by excessive body fat accumulation. It is known to have an adverse effect on health and to lead to reduced life expectancy and several diseases (1,2). Based on independent studies (3,4), 9.1-12.8% of the general population and 3.6% of school children are obese in Turkey. While many studies aiming to clarify the etiopathogenesis of obesity have been conducted, none of them was able to determine a single etiology. Probably, obesity develops through an interaction of both genetic and environmental factors.

In the literature, the vast majority of studies focus on the nuclear genome in order to evaluate the molecular genetic basis of obesity. To date, several genes have been found to be related to obesity including the leptin (LEP) gene on chromosome 7 q, leptin receptor (LEPR) gene on 1 p, pro-opiomelanocortin (POMC) gene on 2 p, melanocortin 4 receptor (MC4R) gene on 18 q, melanocortin 3 receptor (MC3R) gene on 20 q, prohormone convertase 1 (PC1) gene on 5 q, peroxisome proliferator activated receptor gamma 2 (PPARγ2) gene on chromosome 3 p, obesity-associated FTO gene on chromosome 16 q 12.2 and the Niemann-Pick C1 (NPC1) gene on chromosome 18 q 11.2 (5,6,7).

In addition to nuclear genes, mitochondrial genome variations have also been evaluated in obesity since mitochondria play an important role in energy metabolism. Mitochondrial ATPase 6 (mt-ATP6) protein encoded by the mitochondrial genome is a component of a large enzyme called ATP synthase which catalyzes the final step of oxidative phosphorylation (8,9). Since the mt-ATP6 gene [NC_001807.4, (8527 to 9207np)] affects the ATP production in the cell, it may be related to obesity which is a metabolic disease resulting from abnormal energy conversion. On the other hand, mitochondrial electron-transfer chain (ETC) is composed of 4 enzyme complexes (I, II, III and IV). Three of these complexes (I, III and IV) contain subunits encoded by mitochondrial DNA (mtDNA). The mitochondrial cytochrome b (mt-CytB) protein is encoded by the mt-CytB gene [NC_001807.4, (14747 to 15887 np)] and is the only mitochondrial genome-encoded subunit of respiratory complex III which plays an important role in electron transport system. Complex III is localized in the inner membrane of mitochondria.

It is known that the nucleotide sequence of mtDNA evolves at a rate 5-10 times higher than that of nuclear DNA. Thus, the differences in maternally transmitted mitochondrial single nucleotide polymorphisms (SNPs) are proposed to be more functionally significant than those SNPs in the nuclear genome (10). The mitochondrial SNPs are within genes encoding proteins that are subunits in the complexes of ETC and oxidative phosphorylation and thus consequently may affect energy metabolism, resting metabolic rate and respiratory quotient (8,9).

To date, several SNPs have been detected in both the mt-ATP6 and mt-CytB genes and were all reported in the human mtDNA database (MITOMAP) (11). It is well known that synonymous substitutions are silent changes in the genome which do not cause any pathology. However, these substitutions may have negative effects on the accuracy, efficiency, and speed of gene expression. Codons resulting from nucleotide substitutions may also cause protein deficiency. On the other hand, nonsynonymous SNPs that cause amino acid changes are believed to have a large impact on protein functions (12,13). Based on this information, we hypothesize that childhood obesity may be associated with certain mtDNA polymorphisms. In order to test this hypothesis, we studied the variations of mt-ATP6 and mt-CytB genes in 100 obese Turkish Caucasian subjects in a case-control study.

## METHODS

A total of 100 unrelated Turkish Caucasian obese children and 100 age-matched healthy children (ages 6-14 years) from the pediatric outpatient clinic of Akdeniz University Hospital were included in the study. Weight was measured using a digital weighing scale sensitive to the nearest 100 g, with subjects in light clothing and bare-footed. Height was measured using a portable stadiometer to the nearest 0.1 cm with subjects standing bare-footed on a flat surface. Age- and sex-specific international percentile charts of body mass index (BMI), recommended by Cole et al (14) were used to determine obesity and overweight status. Overweight status is defined as a BMI between the 85th and 95th percentile, and obesity as a BMI above the 95th percentile. Healthy subjects with a BMI between the 5th and 85th percentiles were defined as of normal weight (control group). Obese patients with Cushing syndrome, hypothyroidism and also those with dysmorphic features were excluded from the study. Genetic studies were performed at Akdeniz University Hospital laboratory settings. Written informed consent forms were obtained from subjects and their parents and approved by the Local Research Ethics Committee of Akdeniz University.

**Molecular Genetic Analysis**

Genomic DNA was purified from peripheral blood as previously described (15). The mt-ATP6 and mt-CytB amplicons were prepared and DNA sequencing analysis was performed in both forward and reverse directions using the Big Dye Terminator Kit v3.1 (Applied Biosystems) in an ABI 3130 Sequencer (16). To amplify and sequence the mt-ATP6 gene, two PCR reactions were done, each covering distinct regions of the gene (Primer set 1: Forward,5’-CACTGTAAAGCTAACTTAGC-3’; Reverse, 5’-AGAATGATCAGTACTGCGG - 3; Primer set 2: Forward, 5’-CCTTACACTATTCCTCATC-3’; Reverse, 5’-TGAAAACGTAGGCTTGGAT-3’). For the mt-CytB gene, the forward primer 5’-ATAGCCATCGCTGTAGTAT-3’ and the reverse primer 5’- CAATTAGGGAGATAGTTGG-3’ were used to amplify and sequence the gene. The PCR amplification condition consisted of initial denaturation at 95 ˚C for 8 min, followed by 35 cycles at 95 ˚C for 45 sec, 55 ˚C for 45 sec, 72 ˚C for 45 sec and a final extension at 72 ˚C for 5 min. Mitochondrial sequence variations were compared with the reference mitochondrial sequence (NM_000492.2) and mtSNPs were identified by comparison with the revised Cambridge reference sequence (11).

**Statistical Analysis**

Mean and standard deviation (SD) values were calculated for each investigated parameter. Statistical analyses were performed by applying the test of differences between two proportions and independent samples, Mann-Whitney U test, student’s t-test and chi-square test using the SPSS 17.0 program.

## RESULTS

**Figure 1:**
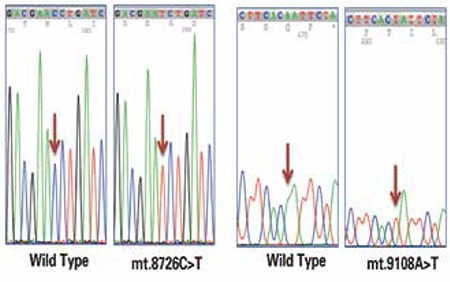
Electropherograms of novel homoplasmic variations detected in the mt-ATP6 gene

**Figure 2:**
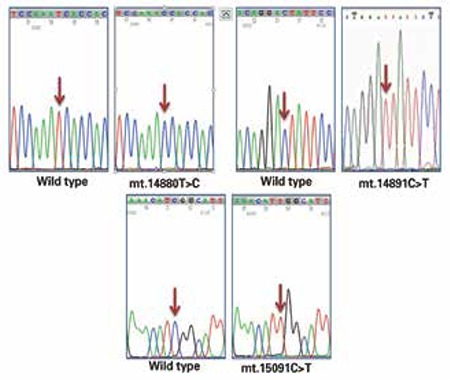
Electropherograms of novel homoplasmic variations detected in the mt-CytB gene

A total of 100 obese and 100 age-matched control subjects were included in the study. Male/female ratio was 41/59 (69.5%) in the obese group and 39/61 (63.9%) in the controls (p>0.05). Thirteen of the obese subjects were the offspring of first-degree consanguineous parents. In the study population (n=200), a total of 118 homoplasmic synonymous and nonsynonymous variations were detected. Based on the overall mitochondrial SNP profile of the mt-ATP6 gene, a total of 51 homoplasmic substitutions (26 nonsynonymous and 25 synonymous) were found. One nonsynonymous substitution (mt.8726C>T) and one synonymous substitution (mt.9108A>T) were novel substitutions which have not been reported in MITOMAP (Table 1). In the mt-CytB gene, a total of 67 homoplasmic substitutions (29 nonsynonymous and 38 synonymous) were found. One nonsynonymous substitution (mt.14880T>C) and two synonymous substitutions (mt.14891C>T and mt.15091C>T) were novel substitutions and have not been reported in MITOMAP (Table 2). Electropherograms of novel variations are shown in Figures 1 and 2. The number of transitions detected in mt-ATP6 (n=49.96%) and the mt-CytB genes (n=65.97%) were higher in both groups compared to the number of transversions in mt-ATP6 (n=2.4%) and mt-CytB genes (n=2.3%). In the obese group, the frequency of mt-ATP6 variations (39/51, 76.5%) was higher than that of the healthy control group (24/51, 47.1%). However, the frequency of SNPs in the mt-CytB gene was similar in both groups: 43/67 (64.2%) in obese group versus 44/67 (65.7%) in controls. The total frequency of nonsynonymous variations was 22.1% (26/118) and that of synonymous variations was 21.2% (25/118) in the mt-ATP6 gene, whereas in the mt-CytB gene the nonsynonymous rate was 24.6% (29/118), and the synonymous variation rate was 32.2% (38/118). The frequencies of each nucleotide substitution (synonymous) and amino acid replacement (nonsynonymous) are listed in Tables 1 and 2. Only two previously reported synonymous substitutions (mt.8614T>C and mt.8994G>A) in the mt-ATP6 gene were found significantly higher in the obese group which were not detected in the control group (p<0.05).

## DISCUSSION

To our knowledge, there is no previous study about the association of mitochondrial variants (mt-ATP6 and mt-CytB) in childhood obesity. To date, several specific mtDNA polymorphisms have been reported from different countries. The first screening study in the mt-ATP6 and mt-CytB genes was done by Fuku in 2002 (17). Fuku and colleagues stated that the mutational strand asymmetry is stronger in the mt-CytB gene compared to the mt-ATP6 gene in 96 young Japanese obese subjects. When they evaluated all of the variations, mt-CytB gene transitions were found at 22 sites accounting for 92% of the variations, whereas transversions found at two sites accounted for 8%. No transversions were detected in the mt-ATP6 gene of the Japanese group. Our data support these findings. In the present study, the transition frequency of mt-ATP6 and mt-CytB genes was approximately 96% and 97% respectively compared to transversions with a frequency of 4% in the mt-ATP6 and 3% in the mt-CytB gene. It is known that a lower transversion rate compared to transitions is a property of mitochondrial genome evolutionary process (18). In our study group, we detected several nonsynonymous and synonymous variations similar to the Japanese obese subjects screened by Fuku et al and Guo et al (Table 3) (17,19). Additionally, we detected a mt.8614T>C substitution in 4 and a mt.8994G>A substitution in 7 obese subjects. None of the control cases have these variations. Therefore, these synonymous substitutions may have a role in the development of obesity through their effects on mitochondrial function. It was shown that one certain codon is more preferential than other codons during protein translation in terms of efficiency of the translation (13). Codon alteration may slow down the formation of ATP synthase resulting in deficient energy and metabolic anomalies. Further large-scaled studies should be done to identify the role of these variations in energy metabolism. In this study, we did not determine a protecting variation for obesity. However, it has been reported that there are some protecting variations in nuclear encoded genes towards obesity, but in mitochondrial genome, it has not yet been identified (20).

In 2003, Okura et al (21) reported that a specific SNP in the mt-CytB gene (mt.15497G>A) may be associated with obesity, body size composition and regional body fat distribution in middle-aged and elderly Japanese subjects, but according to Liguori et al (22), there is no relationship between this polymorphism and obesity in adult Caucasian subjects (Table 3). Previously, it was reported that the A allele of mt.15497G>A may be one of the important determinants of obesity (23). Based on the data by Okura et al (21), this SNP may be more common in people from central Asia and the Arctic region, since this SNP was rarely detected in the Mediterranean Italian population. Interestingly and in support of the above studies, neither our obese nor healthy subjects have this SNP at mt.15497 position.

It was shown that mitochondrial genome variations might affect the metabolic parameters and might have a role in bioenergetics pathways, metabolic rates and energy consumption, depending on ethnic backgrounds (17,19,21,22). It is also well known that mitochondrial metabolism is important for maintaining body temperature for climate adaptation (24). It is suggested that nonsynonymous mitochondrial SNPs may have been selected for adaptation toward an energy efficient metabolism. The present study was performed in a well-defined area localized on the south coast, Mediterranean region of Turkey which has a very hot and humid climate compared to other regions of the country. Therefore, the similarities between our findings and those of the Italian researchers might be due to similarities in the climate.

We should point out that geographically, Turkey has been an area of settlement or the passage route of several Mediterranean, Middle Eastern, and Asian populations in its history. Thus, there is considerable genetic variation among the contemporary population residing in Turkey (25). The variations detected among the present and other studies may be explained by this admixture. Recently, Grant et al (24) have focused on a large cohort study of European-American (n=1080) and African-American (n=1479) obese children versus lean controls. They have concluded that common mitochondrial polymorphisms or heteroplasmy do not play a role in childhood obesity, and no association was detected between childhood obesity and any of the assayed mitochondrial polymorphisms in either ethnicity. Since our study population is focused on Anatolia, which is under the effect of different cultures over the past years, our conclusive data are in line with this recent report.

To our knowledge, there is no report focusing on the relationship between mt-ATP6 and mt-CytB variations and childhood obesity in the relevant literature. As this is an initial study, we conclude that further SNP data should be gathered to highlight the relationship of known mitochondrial variations in addition to novel ones with childhood obesity in specific populations like that of Turkey. In this era of personalized medicine, we believe that knowing the SNP profiles of obesity related to mitochondrial and nuclear genes in specific populations may help to establish the basis of nutrigenomic studies.

**Acknowledgement**

The authors would like to thank Lynn P. Chorich from Institute of Molecular Medicine and Genetics, Georgia Regents University, Augusta, GA, USA for editing the manuscript.

## Figures and Tables

**Table 1:**
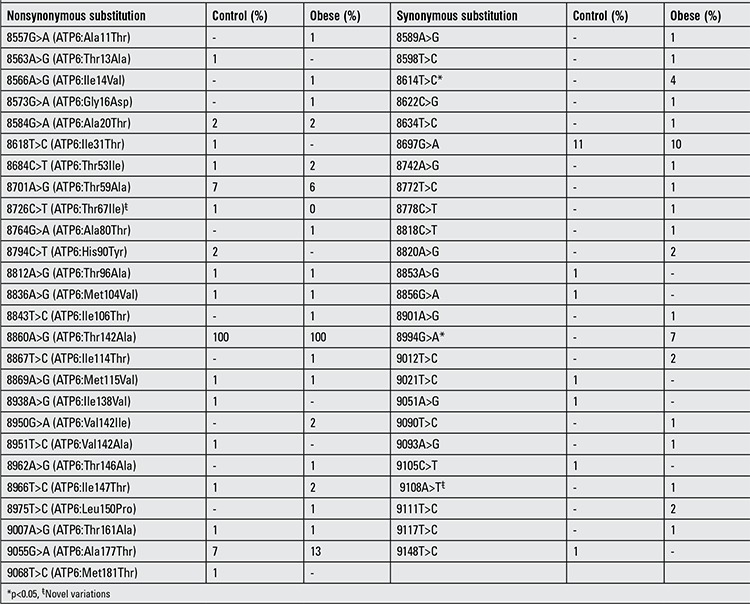
List of nonsynonymous and synonymous substitutions detected in mitochondrial ATPase subunit 6 (mt-ATP6) gene in obese and control subjects

**Table 2:**
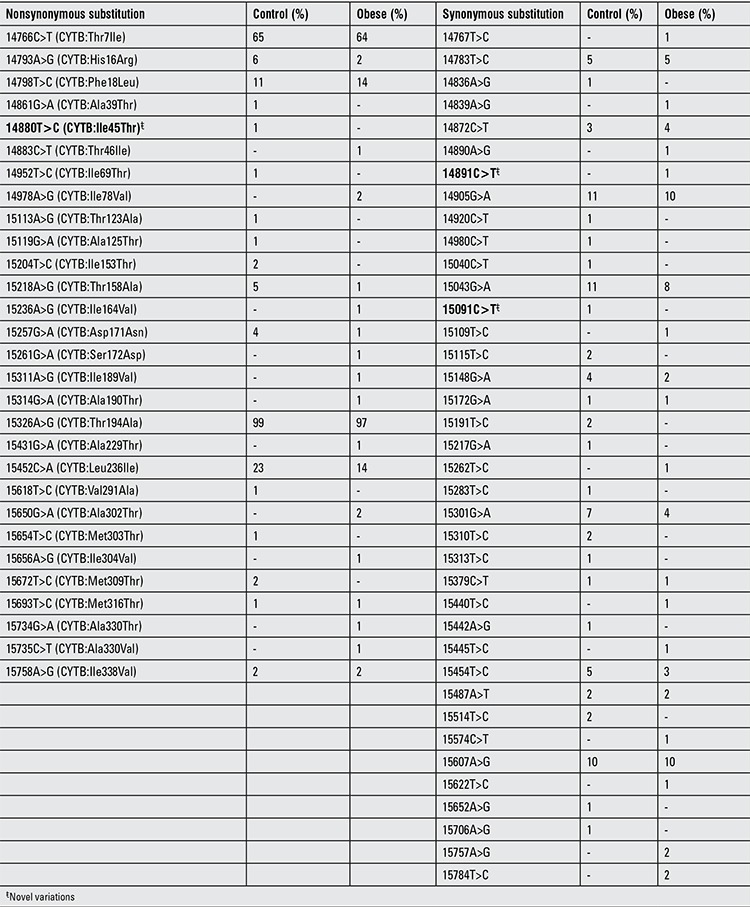
List of nonsynonymous and synonymous substitutions detected in mitochondrial cytochrome B (mt-CytB) gene in obese and control subjects

**Table 3:**
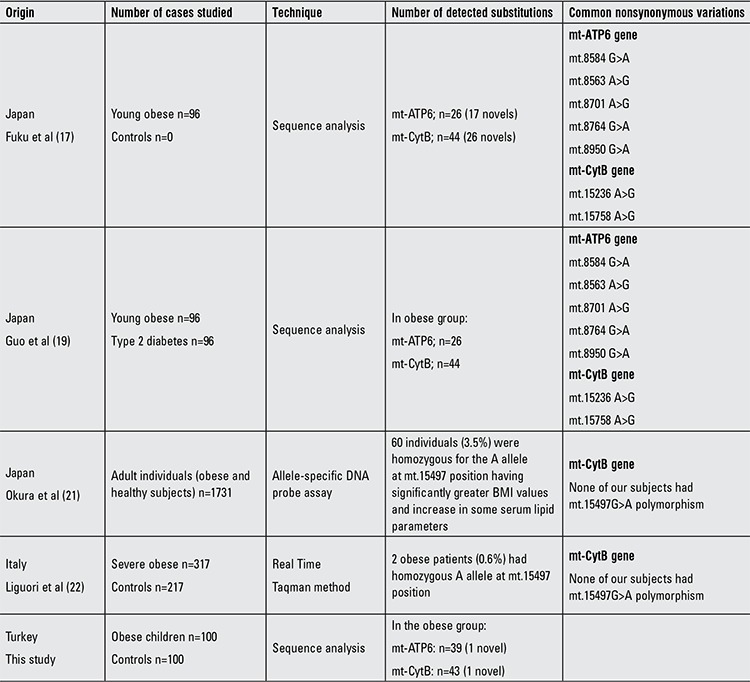
List of common nonsynonymous variations in Caucasian and Asian obese populations
